# Assessing the relationship between atmospheric aerosols and maximum surface air temperature over the Indian region

**DOI:** 10.1038/s41598-026-40641-0

**Published:** 2026-02-18

**Authors:** T. S. Sarin, V. Vinoj

**Affiliations:** School of Earth Ocean and Climate Sciences, Indian Institute of Technology Bhubaneswar (IIT BBS), Argul, Odisha 752050 India

**Keywords:** Atmospheric aerosol, Surface temperature, MERRA2, MODIS, India, Atmospheric science, Atmospheric chemistry

## Abstract

Atmospheric aerosols significantly influence Earth’s climate through direct, indirect, and semi-direct effects on solar radiation. While their global cooling impact at the surface is well-documented, regional-scale studies using observations remain limited. This study quantifies aerosol impact on maximum surface air temperature over India, a region characterised by substantial diversity in aerosol types and seasonality, using long-term satellite observations, reanalysis datasets analysed with a multiple linear regression framework that accounts for cloud cover and atmospheric moisture, and complementary regional climate model simulations to validate the underlying physical mechanisms. Results reveal strong spatial variability in the aerosol effect (AER_EFF_OBS_) with the aerosols appearing to cool the surface by −0.25 °C in winter (DJF) and −0.04 °C in the post-monsoon (SON) while warming it by 0.15 °C in the pre-monsoon (MAM). This warming/cooling appears to be linked to aerosols and aerosol-induced changes in clouds. Analysis using reanalysis data (MERRA2) yields an aerosol effect (AER_EFF_RA_) of −0.45 °C in DJF, 0.18 °C in MAM, and −0.12 °C in SON matching the spatial patterns in AER_EFF_OBS_. Model simulations using the Regional Climate Model (RegCM 4.7.1) further corroborate the effect of aerosol-induced changes in low cloud cover affecting the temperature. These results underscore the intricate relationship between aerosol and surface temperature over India, a region with high aerosol loading, emphasizing the need for improved understanding of aerosol-cloud-climate interaction.

**Supplementary Information:**

The online version contains supplementary material available at 10.1038/s41598-026-40641-0.

## Introduction

Atmospheric aerosols are fine microscopic particles originating from both natural sources such as dust, sea salt, and volcanic activity, as well as from anthropogenic activities including fossil fuel combustion and biomass burning. Although they constitute only a small fraction of atmospheric mass, aerosols significantly influence Earth’s climate by interacting with solar, terrestrial radiation and by modifying cloud properties. They influence the energy balance through direct effects (scattering and absorption of solar radiation^1,2^, indirect effects (modifying cloud microphysics as cloud condensation or ice nuclei^3–5^, and semi-direct effects (altering atmospheric heating profiles and cloud dynamics via absorbing aerosols like black carbon^6^. Together, these aerosol-radiation and aerosol-cloud interactions typically result in a net surface cooling effect at the global scale, although the magnitude and even the sign of the effect can vary substantially across different regions and seasons^7–9^.

Surface air temperature (SAT) is among the most essential variables in climate and meteorological studies, influencing key processes such as atmospheric circulation, moisture distribution and precipitation^10^. Aerosols can modulate SAT by their effects on the energy balance as described above. Several studies have investigated the aerosol-SAT relationship using statistical methods and climate models, with much of the research focusing on the influence of anthropogenic aerosols on global mean SAT^9,11^, generally indicating a net negative radiative forcing at the surface and a positive forcing in the atmosphere For instance, Samset et al^12^., using four fully coupled global atmosphere–ocean–composition models, estimated that anthropogenic aerosols reduce global mean SAT by approximately 0.5–1.1 K. However, aerosols vary significantly in their chemical composition and atmospheric burden across regions and seasons. These variations arise from both local factors (e.g., emission sources, meteorological conditions, land-surface characteristics) and remote influences (e.g., long-range transport, circulation patterns, and large-scale climate variability)^9,13^, all of which shape aerosol-radiation and aerosol-cloud interactions. As a result, global averages can overlook important regional patterns, highlighting the need for region-specific assessments of aerosol impacts on surface temperature at finer spatial and temporal scales. Another key limitation of current literature is the heavy reliance on model-based assessments to quantify aerosol impacts on SAT. While climate models have improved in capturing the direct radiative effects of aerosols, they continue to struggle with representing the indirect and semi-direct effects, which involve complex aerosol–cloud–radiation interactions^14^. These processes can even exceed the magnitude of direct effects, and their omission or oversimplification can lead to incomplete or biased interpretations (IPCC, 2014). This highlights the importance of integrated approaches that combine model simulations with observational constraints. Although long-term in-situ measurements are limited, the advent of high-quality, continuous satellite observations since the early 2000s offers a promising opportunity to study aerosol–SAT interactions at regional and seasonal scales with improved spatial resolution.

Among global regions with substantial aerosol loading, such as India, China, and West Africa, each exhibits distinct seasonal and chemical characteristics. In West Africa, for instance, desert dust and biomass-burning aerosols dominate, while in China, a combination of industrial emissions and natural sources contributes to high aerosol burden^15–19^. India presents a unique mix of aerosol types from both anthropogenic and natural sources. Northern India, in particular, has some of the highest population densities and industrial activity in the world, leading to high levels of pollution and aerosol loading^20^, which becomes especially higher during the winter season due to the stagnant conditions that prevail over the region as well as seasonal crop burning practices^21^. In addition, the region is also strongly influenced by desert dust transport from the Thar and the Arabian desert^22,23^. The monsoon season is characterised by the large-scale transport of sea-salt aerosols from the Arabian Sea onto the subcontinent^24,25^, while post-monsoon biomass burning in the region further enhances aerosol concentrations^21^. This complex and seasonally varying combination of sources makes India a particularly relevant region for investigating the climatic role of aerosols.

Previous studies over the Indian region have suggested that aerosols can substantially alter the surface energy balance by reducing incoming solar radiation while enhancing atmospheric longwave radiation, with the net effect being cooling or warming depending on aerosol type and concentration^26–28^. The sharp reduction in emissions during the COVID-19 lockdown further illustrated this relationship, as satellite observations revealed a rise in daytime surface temperatures across multiple land and oceanic regions, including India^29–32^. Aerosol-SAT relationships have also been studied extensively in the context of monsoon variability, where aerosols are known to weaken the north–south temperature gradient that drives large-scale circulation, mainly by reducing surface-reaching solar radiation and cooling the land surface^28,33–35^ . Absorbing aerosols, such as black carbon, add further complexity by heating the atmosphere while cooling the surface, potentially altering vertical stability, shifting circulation patterns, and even warming remote regions in ways that offset local cooling^26^. At the regional scale, Roy^27^ reported that aerosols exert neutral to warming effects during summer but contribute to widespread surface cooling in winter across India, while others have linked aerosol loading to shifts in temperature ranges and diurnal variability^36^. More recently, absorbing aerosols such as black carbon and dust have been shown to intensify heat waves^37,38^, and aerosol-induced temperature perturbations have been associated with reductions in agricultural productivity, particularly in crops such as wheat^39,40^.

Together, these studies underscore the complex and far-reaching impacts of aerosols. However, most existing research has concentrated on urban settings or isolated climate events, providing limited insight into broader seasonal and regional climatology/variability across the Indian subcontinent. To address this gap, the present study uses satellite-based observations over India with two primary objectives: (1) to explore and develop a method for estimating changes in maximum surface air temperature associated with aerosol loading, while controlling for confounding variables such as cloud cover and water vapor; and (2) to examine the spatial and seasonal variability of aerosol-temperature relationships across India. These objectives are designed to advance understanding of aerosol-induced surface temperature changes in a region characterised by high aerosol concentrations and pronounced climate sensitivity. It should be noted that the monsoon season was excluded from this analysis due to the complex interactions among aerosols, precipitation, and large-scale circulation, which can obscure direct aerosol-temperature relationships and are beyond the scope of the present study.

## Results and discussion

### Observational analysis of aerosol effect

**Fig. 1:**
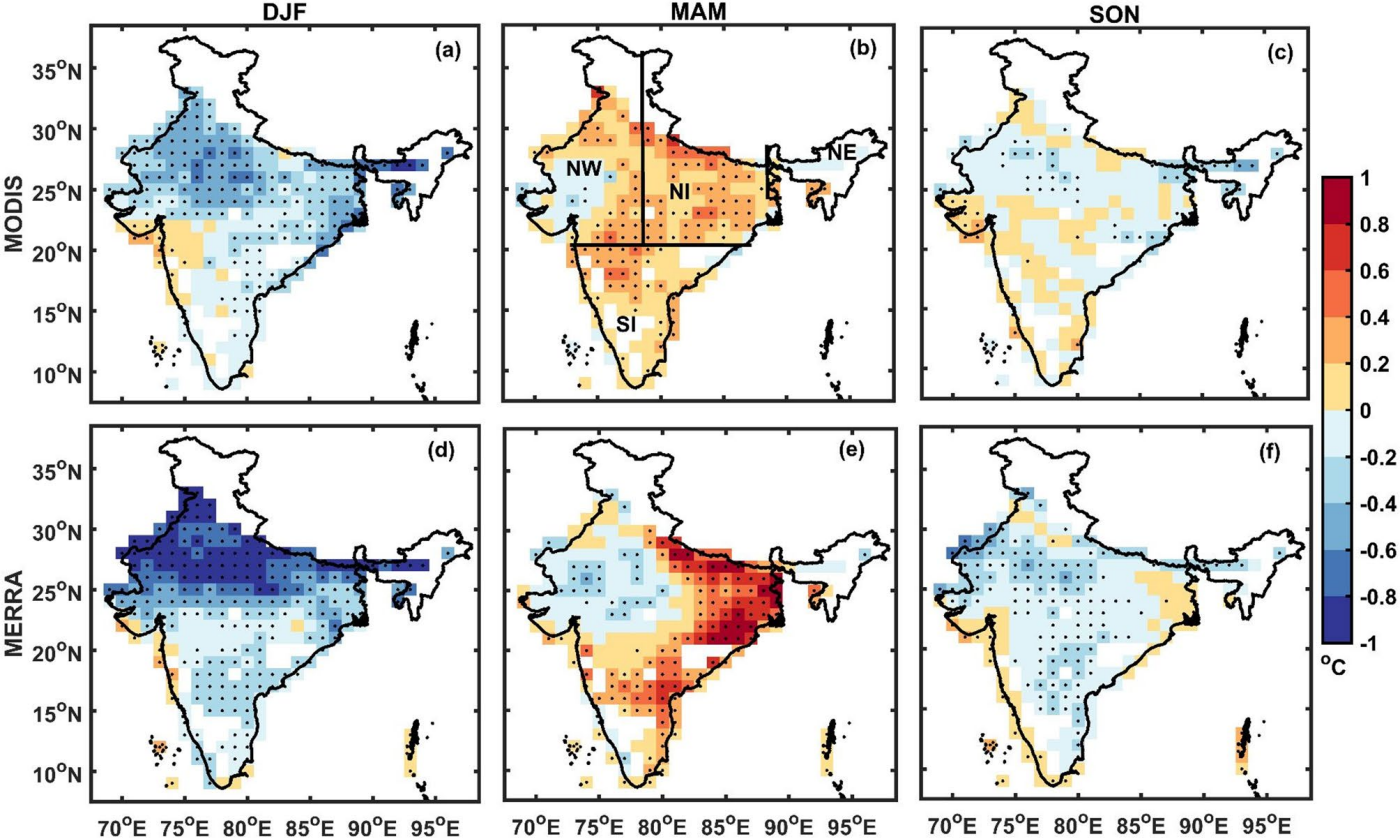
Spatial distribution of the aerosol effect (°C). Dots indicate grid points statistically significant at the 95% confidence level. Panels (**a**–**c**) show AER_EFF_OBS_ estimates for DJF, MAM, and SON, respectively. Panels (**d**–**f**) show corresponding AER_EFF_RA_ estimates. Regional boundaries referenced in the text i.e. Northwest India (NW), North India (NI), South India (SI), and Northeast India (NE) are also marked in panel (**b**). Figure was generated using MATLAB (version R2022b; https://www.mathworks.com).

Figure 1 illustrates the seasonal influence of aerosols on surface air temperatures across India. We apply a multiple linear regression approach (refer to Data and Methods) that accounts for cloud cover and atmospheric moisture to isolate the aerosol direct and semi-direct effects, using both satellite observations and reanalysis data. The results reveal a consistent pattern of aerosols cooling the surface during winter (DJF) and post-monsoon (SON), while leading to surface warming in the pre-monsoon season (MAM) across regions. AER_EFF_RA_ closely mirrors the AER_EFF_OBS_, capturing both the timing and spatial extent of temperature responses. Across these patterns, AER_EFF_OBS_ and AER_EFF_RA_ are generally stronger over northern India, consistent with the region’s higher aerosol concentrations. In the sections that follow, we delve deeper into these seasonal and regional patterns, compare observational and model results, and explore the underlying physical drivers, with a focus on cloud-aerosol interactions.

During the winter season (DJF), aerosols exert a significant cooling effect across most of India, with estimated reductions in maximum surface air temperature ranging from approximately − 0.2 °C to − 0.6 °C (Fig. 1a). The largest cooling is observed over northwestern and northern India, including Rajasthan, Punjab, and Haryana (greater than − 0.5 °C in some regions). Central and southern India also experience notable cooling (− 0.2 to − 0.4 °C), while the effect is relatively weaker in the eastern Indo-Gangetic Plain (IGP), where cooling is closer to − 0.2 °C. Overall, these results suggest that aerosols consistently cool the surface across India during DJF, which may exacerbate the stagnant meteorological conditions during the season and reduce vertical mixing, leading to pollutant accumulation which can further lead to more cooling^41^.

While one might expect stronger cooling over regions with high aerosol concentrations, such as the IGP, our analysis shows that this relationship is not straightforward. During DJF, the IGP has the highest concentration of both scattering aerosols like sulphates and carbonaceous aerosols like black carbon and organic carbon which are absorbing in nature^42^. The relatively weaker cooling over IGP could result from offsetting effects between the two types of aerosols, which modulate the net radiative forcing at the surface. This mismatch between high aerosol concentrations and the simulated cooling response highlights the importance of not just aerosol abundance but their composition. A localized warming anomaly is seen along the western coast near Mumbai, potentially linked to high concentrations of black carbon associated with the Bombay Plume^43^, which, through semi-direct effects, could reduce cloud cover and enhance surface reaching solar radiation^44,45^. However, this is an exception within a broader pattern of cooling.

These findings align with the modeling-based study by Freychet et al.^46^, who reported similar spatial patterns of aerosol-induced cooling using the HadGEM3-GA6 model nudged to ERA-Interim data. Similar wintertime aerosol induced surface cooling has also been reported by Kitabayashi and Takahashi^47^.The present results, based entirely on observational and reanalysis datasets, offer a data-constrained view of aerosol effects, providing robust, large-scale evidence of wintertime surface cooling attributable to aerosols.

During the pre-monsoon (MAM) season (Fig. 1b), aerosols are associated with widespread surface warming across much of India. This effect is particularly pronounced over northern India (NI), where average warming reaches approximately + 0.23 °C, with localized increases from + 0.2 °C to + 0.8 °C and exceeding + 1 °C in some regions. In contrast, parts of northwestern India exhibit isolated pockets of cooling. This spatial pattern indicates that the commonly held assumption linking increased aerosol loading to surface cooling does not always hold. These observations align with Roy^27^, who reported neutral to positive temperature responses during the pre-monsoon season due to enhanced longwave radiation under overcast conditions. The persistence of warming in our results, even after accounting for cloud cover, suggests additional mechanisms involving the absorbing aerosols and regional meteorological conditions. Another study by Jangid et al.^48^ estimated a negative aerosol-induced surface forcing (−1.30 °C ± 0.18 °C) over the Indian Core Monsoon Region (ICMR) during the pre-monsoon season. However, their analysis focused on clear-sky conditions for isolating aerosol effects on SAT, though non-removal of seasonal and long-term trends from the data may have biased the results.

One plausible reason for the warming is the substantial presence of absorbing aerosols, primarily mineral dust, in the lower troposphere over northwestern India during this period^37,49,50^. Coarse-mode aerosols, such as dust, which significantly contribute to longwave (LW) warming, are underrepresented in satellite retrievals and reanalysis datasets^51^, suggesting the observed warming could be underestimated. Aerosols influence surface temperatures through intrinsic LW radiative effects, enhancing downwelling LW radiation while reducing outgoing LW radiation, particularly under heavy aerosol loading^37,38,52–54^. Although secondary to shortwave (SW) effects, LW contributions become significant in highly polluted or dust-laden environments. Black carbon emissions from densely populated regions of the Indo-Gangetic Plain (IGP) may also enhance warming through similar absorption-driven processes. Surface characteristics, particularly land cover, further modulate the aerosol-temperature relationship^55^. For instance, small concentrations of absorbing aerosols over highly reflective surfaces can produce net positive radiative forcing, leading to warming^1^. However, intrinsic LW effects and surface albedo alone cannot fully explain the observed warming, as aerosols cool near primary dust source regions in northwestern India^56^, while warming occurs farther from these sources.

Elevated aerosols transported from source regions may also modify cloud characteristics, particularly vertical distribution, contributing to local energy imbalances. Although individual dust particles are weak SW absorbers, their atmospheric mass can produce significant warming^57,58^. Additionally, dust absorbs in the infrared and LW spectra due to its mineralogical composition^59^, potentially enhancing lower-atmosphere warming and influencing cloud formation at different altitudes. This hypothesis is further explored in subsequent sections, where we assess the role of cloud height in modulating aerosol impacts on surface air temperature.

Cloud height plays a crucial role in determining the net surface energy balance with low-level clouds having a higher potential to cool the surface by reflecting incoming solar radiation compared to higher level clouds^60^. Aerosols, especially, absorbing aerosols at elevated layers may contribute to the dissipation or "burn-off" of low-level clouds, thereby allowing more solar radiation to reach the surface and enhancing surface warming^61^). Bollasina and Nigam^62^ noted that excessive aerosol loading in the pre-monsoon season can reduce total cloud cover, increase surface shortwave radiation, and lead to land surface warming. Similar mechanisms were observed in RegCM simulations by Das et al.^63^ for the monsoon season, characterized by comparable aerosol profiles to the pre-monsoon period, where warming patterns were attributed to a combination of cloud burn-off, and the relative dominance of absorbing over scattering aerosols which counteracted the direct aerosol dimming. These findings suggest that heavy loading of absorbing aerosols may offset the expected cooling from clouds and scattering aerosols through semi-direct effects. The warming induced by this mechanism is likely amplified in regions with strong radiative heating due to elevated aerosol layers^64^. However, the lack of a warming signal over northwestern India (Fig. 1b and e), despite it being a major dust source, indicates that aerosol-induced changes in cloud vertical structure may be more critical than aerosol loading alone in determining the sign of the aerosol-temperature relationship. Notably, this warming signature is largely absent during other seasons, likely due to reduced loading of dust/absorbing aerosols.

The seasonal dependence of the aerosol effect becomes even more apparent during SON (Fig. 1c), which shows a relatively weaker cooling influence compared to DJF approximately between + 0.2 °C and −0.2 °C, likely due to the reduced aerosol burden following the monsoon washout. As in DJF, aerosols contribute to surface cooling across India during SON, albeit at a smaller magnitude potentially reinforcing stable atmospheric conditions and reducing vertical mixing, which may enhance pollutant accumulation and further amplify cooling effects. Notably, the progression from moderate cooling in SON to stronger cooling in DJF, followed by a warming effect in MAM, highlights a clear seasonal pattern. This transition may be driven by increasing concentrations of absorbing aerosols, especially dust, during the pre-monsoon compared to the winter season, which can offset or even reverse the typical surface cooling associated with aerosols under certain conditions. Such seasonal shifts underscore the importance of aerosol type, loading, and atmospheric interactions in determining their net radiative effect.

This seasonal pattern in aerosol-induced temperature changes i.e. cooling during DJF and SON and warming during MAM, is further validated using MERRA reanalysis data (Fig. 1d–f). The aerosol effect estimated from MERRA exhibits similar spatio-temporal behavior as observed in satellite-based results, reinforcing the robustness of the findings. While both datasets agree on the broad seasonal trends, MERRA-derived effects tend to be slightly stronger during DJF though the spatial patterns remain similar. During MAM, the estimates are comparable, with MERRA showing a modest expansion of cooling regions in northwestern India. In SON, spatial consistency is particularly strong over northern India. This difference likely arises from variations in the representation of aerosol radiative properties, limitations in MODIS temperature retrievals under high aerosol or cloud conditions, and inherent differences between reanalysis and satellite datasets due to model physics and data assimilation schemes. Despite these discrepancies, the overall spatial and temporal patterns remain consistent across both datasets confirming that reanalysis data can reliably reproduce the aerosol effects captured via satellite observations.

**Fig. 2:**
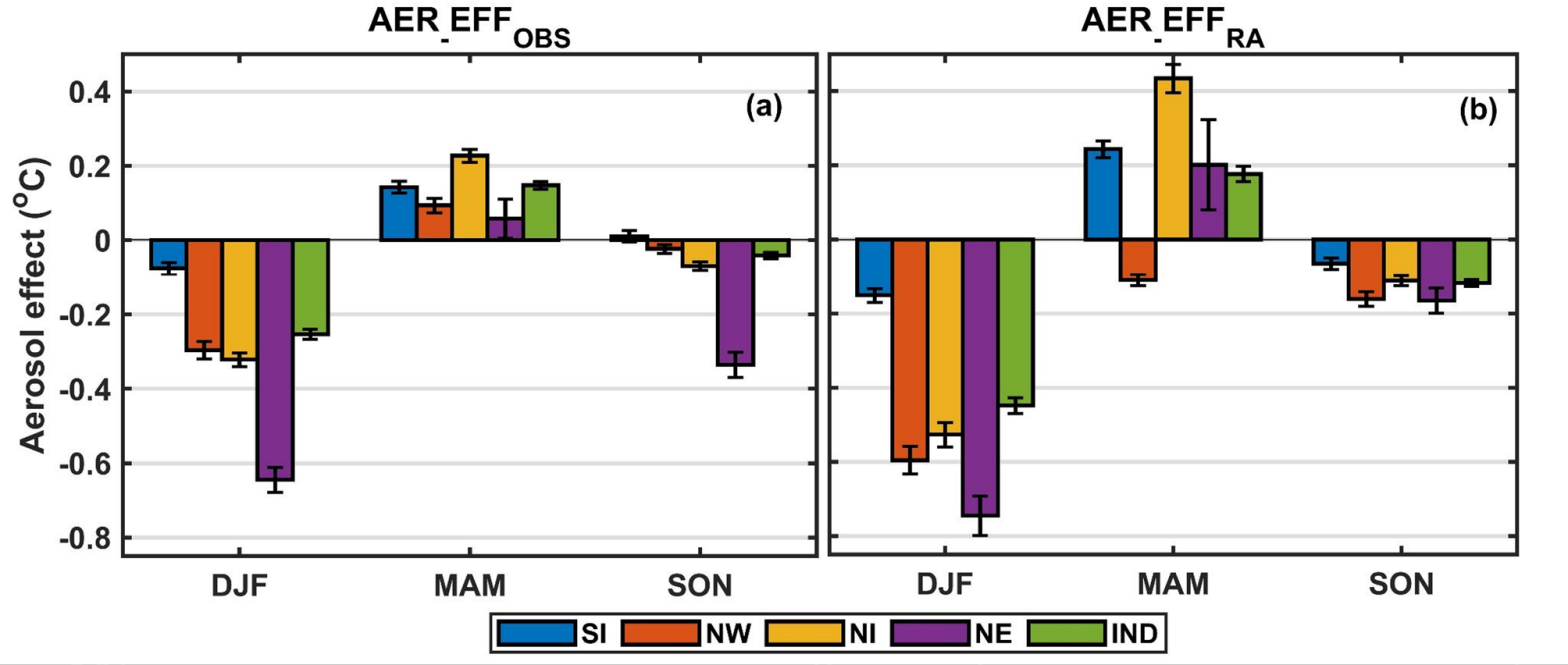
Average aerosol effect over different regions of India (regions marked in Fig. 1b) (**a**) MODIS (**b**) MERRA. The bars denote the standard error of the effect in the particular region.

Figure 2 shows the regionally averaged AER_EFF_OBS_ and AER_EFF_RA_ (regions marked in Fig. 1b). Most of Northern India exhibits statistically significant and strong AER_EFF_OBS_ and AER_EFF_RA_ compared to SI. This spatial variation aligns well with aerosol loading patterns, which are generally higher over northern India. For AER_EFF_RA_, nearly all regions show a stronger aerosol effect (in magnitude) during DJF compared to AER_EFF_OBS_. The warming patterns observed during MAM are also consistent across both datasets, except in northwestern India, where MERRA indicates a more pronounced aerosol cooling effect (compare NW in Fig. 2a and b)*.* During SON, aerosol effects remain negative across most of India, except southern India (SI). Despite some regional variability, both satellite and reanalysis-based analyses demonstrate consistency in the sign of aerosol effects across all seasons and regions. In addition to the seasonal patterns described above, a two-epoch comparison (2002–2013 vs. 2014–2024) confirms that these aerosol-temperature effects are temporally robust, with their magnitudes strengthening in recent years, manifesting as enhanced DJF cooling, intensified MAM warming, and widespread SON cooling (Supplementary Fig. S6).

These findings underscore the importance of advancing our understanding of how aerosols influence surface temperature. It may be noted that our regression analysis, incorporating total cloud cover as a covariate, suggests that variations in cloud fraction alone may not explain the contrasting aerosol effects observed during the pre-monsoon and winter seasons. Instead, recent studies point to a more complex mechanism involving the critical role of cloud vertical structure and type in regulating the surface energy budget^65^. Elevated layers of absorbing aerosols, such as dust and black carbon, can alter atmospheric stability and suppress low-level cloud development, thereby enhancing surface warming via the semi-direct effect^6,61^. Conversely, under certain thermodynamic conditions, aerosols may invigorate convection or enhance low-level cloud cover, leading to greater reflection of solar radiation and surface cooling[100].

Given that most aerosols reside within the lower troposphere, their interactions with clouds are expected to predominantly influence low-level cloud formation and properties. Changes in low cloud cover are particularly relevant for surface temperature regulation, as these clouds exert a strong shortwave cooling effect by reflecting incoming solar radiation. Therefore, it is felt that an investigation on low-level clouds is essential for understanding the mechanisms driving the seasonal variability in aerosol-induced surface temperature responses. To explore this, we assess the impact of aerosol–cloud interactions on both cloud height and low-level cloud cover using observational datasets and regional climate simulations from RegCM version 4.7.1. This analysis aims to clarify how changes in clouds, especially at lower levels, mediate the surface temperature response to aerosol forcing. Details of the model configuration and experimental design are provided in the Data and Methods section (Table 1).

**Table 1:** Model configuration used.

Model used	RegCM (version 4.7.1)
Grid dimensions	96 × 96, 18 sigma levels, centred at 23^o^N 82^o^E
Dynamics	MM5 hydrostatic
Horizontal resolutions	50 km
Top layer pressure	50 hPa
Land surface model	CLM4.5^66^
Meteorological boundary conditions	ERA-Interim 75
Chemical boundary conditions	AERO
Cumulus convection scheme	Emmanuel over land and Grell over ocean
Radiation scheme	CCM3
Moisture scheme	SUBEX
Planetary boundary layer scheme	Holtslag
Topography	USGS
SST	ERA-Interim 75
Tracers	DUST4 (4 bins) SSLT (2 bins) BC + OC (hydrophilic and hydrophobic), Sulphate (2)
Indirect effect	Not considered
Domain	South Asia CORDEX domain
Moisture	ESACCI
Emissions	IIASA

### Aerosol induced changes in low-clouds and its effect on surface air temperature

Despite known limitations in aerosol representation, particularly for anthropogenic sources, RegCM 4.7.1 remains a computationally reasonable and valuable tool for investigating aerosol-induced temperature changes. The simulations and analysis were designed to elucidate the underlying physics, particularly the semi-direct effect and its effect on low cloud levels on the observed aerosol effects (Supplementary Fig. S1). Overall, the model reproduces key spatial and temporal aerosol patterns reasonably well (Supplementary Figs. S4 & S5), with biases likely arising from the use of coarse-resolution emission inventories^67,68^ and simplifications in atmospheric chemistry representation, as noted in previous studies^69^ . Such biases, while common in regional climate modeling, highlight the inherent challenges in accurately capturing aerosol processes at fine spatial and temporal scales.

The simulated aerosol radiative effect at the surface is negative, indicating the dominant role of aerosol direct effect in cooling the surface. However, the presence of both warming and cooling on surface air temperature suggests the influence of other mechanisms like the semi-direct effect, in modulating surface temperature. Therefore, focusing on regions and periods with contrasting aerosol effects on SAT in the entire simulated domain (at each grid point), thus offers a useful framework for interpreting the observed dichotomy in satellite-derived aerosol–temperature relationships.

**Fig. 3:**
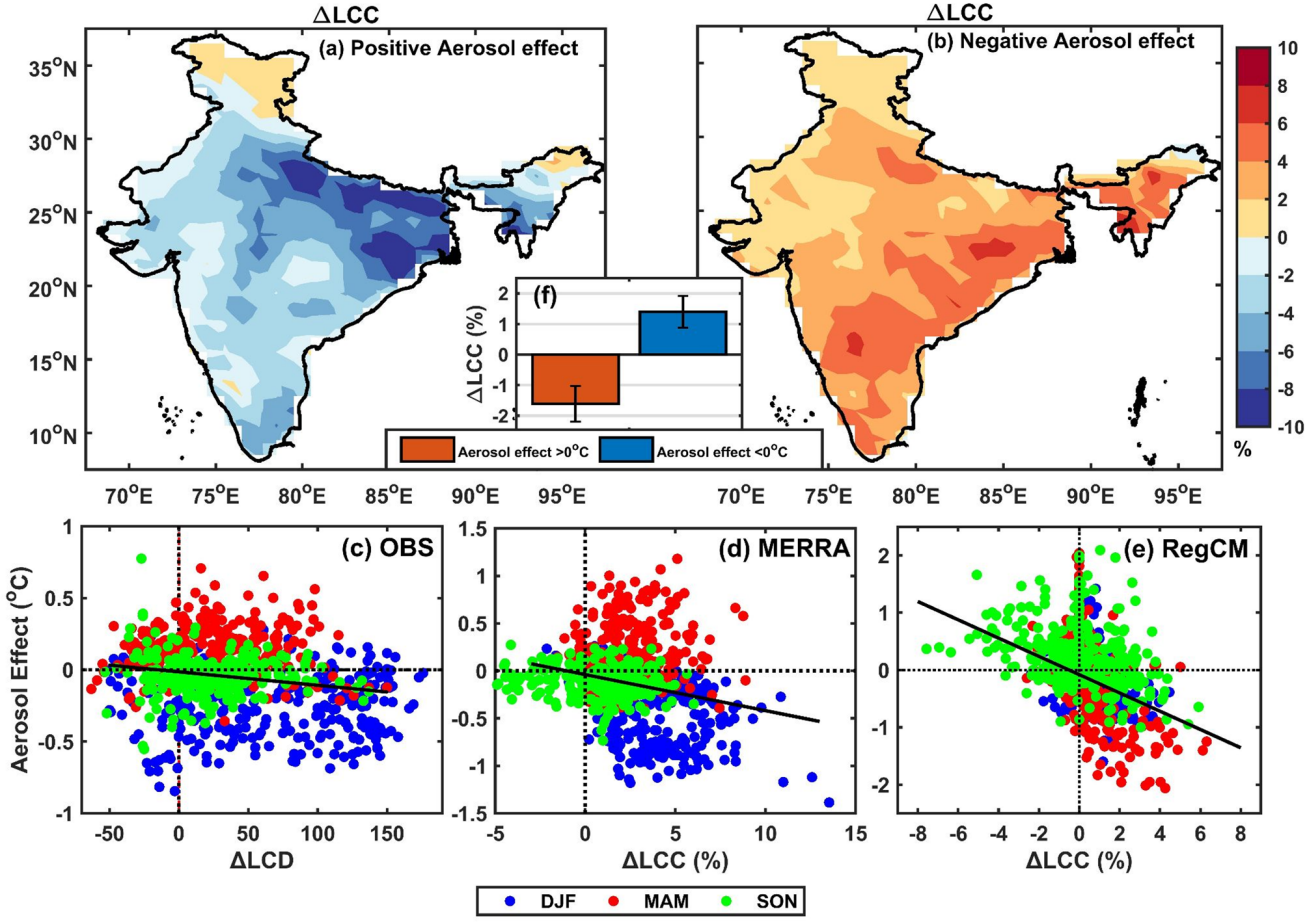
(**a**) Aerosol-induced change in low cloud cover when the aerosol effect is positive (**b**) Aerosol-induced change in low cloud cover when the aerosol effect is negative (**c**) Aerosol effect v/s Aerosol-induced change in low cloud days (**d**) Aerosol effect v/s Change in low cloud cover in RegCM (**e**) Average change in low cloud cover in two aerosol effect regimes. Figure was generated using MATLAB (version R2022b; https://www.mathworks.com).

Figure 3a,b illustrates aerosol-induced changes in low cloud cover (ΔLCC_MOD_), categorized by two surface temperature response regimes: periods with aerosol effects below 0 °C (cooling) and those above 0 °C (warming) in the whole year long simulation. During times associated with a negative aerosol effect, cloud cover increases modestly across all levels, typically ranging from 0 to 8%. In contrast, periods with a positive aerosol effect show a more pronounced decrease in cloud cover, reaching up to 10%. These patterns are consistent with the semi-direct effect, wherein aerosol-induced atmospheric heating modifies the vertical temperature profile, thereby suppressing or enhancing cloud formation^6^. Such aerosol-cloud interactions likely play a significant role in shaping the net surface radiative balance, and in turn, determine the sign and strength of the aerosol impact on surface air temperature.

One plausible explanation thus, for the observed warming despite negative surface radiative forcing is the aerosol-induced burn-off of low-level clouds. Previous studies (e.g^11,44,45^.) suggest that absorbing aerosols can heat the surrounding atmosphere, destabilize the lower troposphere, and lead to cloud dissipation. This reduction in cloud cover allows more solar radiation to reach the surface, enhancing warming. This mechanism helps to reconcile the observed spatial heterogeneity in aerosol effects, particularly in regions showing a positive surface temperature response. To assess whether this mechanism holds in real-world conditions, we next examine satellite observations and reanalysis data for evidence of aerosol-induced cloud changes mediating the aerosol-surface air temperature relationships.

Figure 3c presents aerosol-induced changes in the number of days with low cloud cover (ΔLCD_OBS_), analyzed against AER_EFF_OBS_ (bottom left). The analysis reveals a clear negative relationship between ΔLCD_OBS_ and AER_EFF_OBS_. The same negative relationship can be seen between AER_EFF_RA_ and ΔLCC_RA_ (Fig. 3d) and between AER_EFF_MOD_ and ΔLCC_MOD_ (Fig. 3e). A regional average of ΔLCD_OBS_ and ΔLCD_RA_ after segregation by the sign of the AER_EFF_OBS_ and AER_EFF_RA_ (Supplementary Table S1) further shows that regimes with negative aerosol effect (surface cooling) generally exhibit larger ΔLCC_OBS_ over India. This is consistent with modelling results (Fig. 3a,b,f) which segregates ΔLCC_MOD_ according to the sign of AER_EFF_MOD_ and finds that a negative AER_EFF_MOD_ is associated with a positive ΔLCC_MOD_ and vice-versa.

**Fig. 4:**
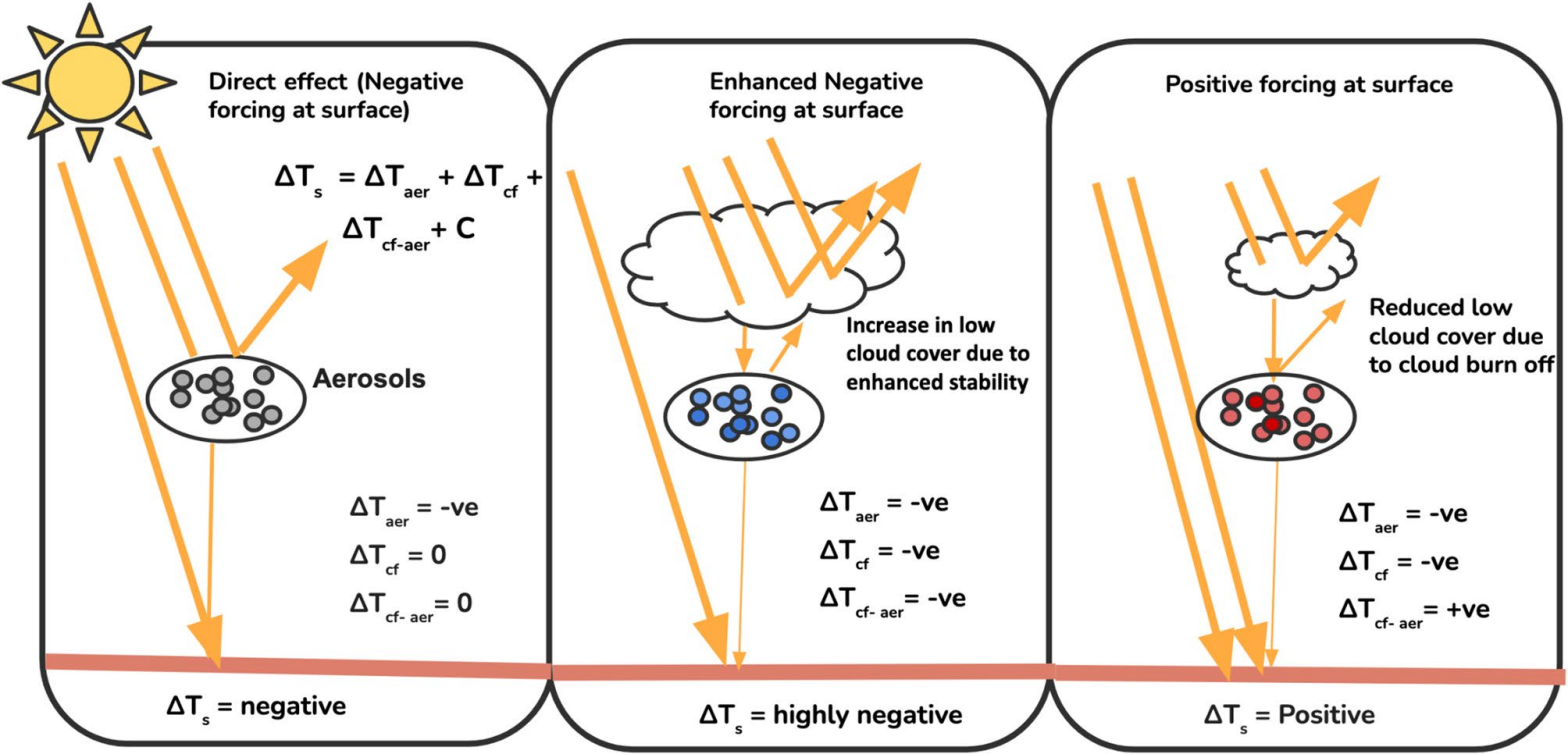
Schematic illustration of three primary aerosol-induced pathways affecting surface temperature. Left: The direct radiative effect of aerosols, where scattering and absorption reduce the net solar radiation reaching the surface. Middle: Cloud invigoration, where aerosols enhance atmospheric stability and increase low cloud cover, leading to higher reflection of solar radiation and surface cooling. Right: Cloud burn-off effect due to absorbing aerosols (e.g., black carbon, dust), which heats the atmosphere, suppresses cloud formation, and increases solar radiation reaching the surface, thereby enhancing warming.

Seasonal patterns further support this relationship. During DJF, aerosols are predominantly linked to increased low cloud days, consistent with the cooling of lower atmospheric layers that stabilizes the boundary layer and promotes low-level cloud formation. These clouds enhance the reflection of incoming solar radiation, amplifying the cooling effect at the surface (Fig. 4, Middle) compared to the case where only the direct effect of aerosol is present (Fig. 4, left). In contrast, the MAM season shows a widespread decrease in low cloud days, particularly in regions dominated by absorbing aerosols such as black carbon and dust. These aerosols heat the atmosphere via the semi-direct effect, suppressing cloud formation by destabilizing the vertical thermal structure and promoting cloud burn-off. This reduction in cloud cover allows greater solar radiation to reach the ground, leading to a positive aerosol effect on surface temperature (Fig. 4, Right) compared to the case where aerosol directly interacts with the radiation (Fig. 4, Left).The SON season emerges as a transitional period, showing a mixed response pattern, gradually shifting from the cloud-enhancing conditions of DJF to the cloud-suppressing regime characteristic of MAM. This seasonal contrast points to a layered mechanism: in DJF, increased low clouds reinforce surface cooling, while in MAM, the burn-off of low clouds (reducing their strong shortwave cooling effect) contributes to a net warming at the surface. Model experiment (Fig. 3e) results support these observations, showing that a positive aerosol effect during all seasons is consistently associated with a decrease in low cloud cover. This alignment between modeled and observed patterns underscores the complex interplay between aerosol-cloud interactions and their impact on surface air temperature and reinforces the need to account for both vertical cloud distribution and regional aerosol characteristics when assessing aerosol impacts on surface temperature.

These observational and model-based findings also align with prior studies that emphasize the critical role of aerosol–cloud interactions in modulating surface temperature. Ackerman et al.^61^, using INDOEX data and large-eddy simulations, demonstrated that absorbing pollution aerosols accelerate the dissipation of trade cumulus clouds via solar heating, thus reducing cloudiness and warming the surface. Lohmann and Feichter^70^ similarly showed that the semi-direct effect of aerosols can reduce both cloud cover and liquid water path, influencing radiative balance. Koch and Del Genio^44,45^ highlight that the net effect of aerosols on cloud properties depends on the aerosol type and its vertical location relative to clouds. Additionally^18,19^, note that clouds can amplify the surface warming associated with absorbing aerosols by enhancing positive forcing and weakening negative forcing at the top of the atmosphere. Together, these studies support the interpretation that aerosol-induced changes in cloud cover, especially reductions driven by absorbing aerosols, are a key mechanism shaping the sign and magnitude of aerosol effects on surface temperature.

Taken together, the observational and modelling analyses underscore that aerosol-cloud interactions play a critical role in modulating surface air temperature responses across India both spatially and temporally. These interactions, particularly through changes in cloud vertical distribution contribute to the observed seasonally varying aerosol effects, highlighting the complex pathways through which aerosols influence near-surface climate. Building on these insights, the study concludes that aerosols exert a significant influence on surface air temperature over India. Understanding how aerosols modify surface temperature is vital for anticipating and mitigating their effects in diverse sectors such as agriculture, solar energy generation, and human health and comfort. For instance, Northern India, already burdened with high pollution levels during the winter, experiences enhanced surface cooling from aerosols, which can intensify atmospheric stagnation and worsen pollution episodes. Conversely, during the pre-monsoon season, aerosol-induced warming alters the land-sea thermal contrast, a key driver of the Indian summer monsoon. This warming is likely to advance the monsoon onset, increase pre-monsoon rainfall, and potentially suppress rainfall during the core monsoon season, posing risks to food and water security across the region. Additionally, aerosols may enhance heat-wave conditions during this period with implications for public health and well-being. Overall, this study highlights that aerosols can either warm or cool the Earth’s surface, depending on their interactions with clouds, specifically low clouds. These aerosol-cloud interactions may help explain the seasonal variations in surface temperature, including cooling and potentially higher air pollution during winter, and warming with potentially more intense heat wave activity during the pre-monsoon, both amplified by positive feedback.

In addition to offering a physical understanding of aerosol-induced temperature changes, this study provides observational benchmarks that can help validate climate models. The strong agreement between satellite observations and MERRA reanalysis data highlights the ability of reanalysis products to reliably capture aerosol effects over the Indian region. Our results show that a model including only the direct and semi-direct effects of aerosols can explain much of the observed surface temperature response. This suggests that these effects play a dominant role in shaping temperature patterns across India. Given the region’s high aerosol levels and complex meteorology, continued investigation into aerosol-cloud-climate interactions including the aerosol indirect effects are essential. Advancing this understanding will be key to improving future climate projections and guiding adaptation efforts in a warming world.

## Data and methods

### Data

Daily datasets for aerosol optical depth (AOD), cloud fraction (CFR), total column water vapor (TCWV), cloud top pressure (CTP), and maximum surface air temperature (T_max_), along with corresponding reanalysis variables were used in this study. All datasets were analyzed at a spatial resolution of 1° × 1°, covering the period from September 2002 to November 2024. This time frame was selected to ensure both data availability and consistency across all variables.

The AOD data is from the MODIS Level 3 (MYD08_D3 v6.1^71^) product aboard the NASA Aqua satellite, obtained via the NASA Giovanni archive (https://giovanni.gsfc.nasa.gov/giovanni/). This dataset combines the Dark Target and Deep Blue algorithms based on surface NDVI, chosen for its broad applicability across diverse Indian surfaces and strong validation performance^72^. Aqua’s overpass time of approximately 1:30 PM local time aligns well with the timing of maximum temperature (~ 2 PM), making it particularly suitable for examining daytime aerosol radiative effects.

CFR and TCWV data is from the AIRS Level 3 product (AIRSX3STD V007), also onboard Aqua^73,74^, accessed through the NASA Giovanni archive. These datasets have been shown to agree well with radiosonde observations^75–77^. Additionally, daily gridded CTP data from MODIS Aqua was used to examine the vertical structure of clouds.

Daily maximum surface air temperature (T_max_), measured at 2 m above ground level, was sourced from the Indian Meteorological Department (IMD) at 1° × 1° resolution. This dataset interpolates measurements from 395 weather stations across India using a modified Shepard’s angular weighting method^78^. Since the study focuses on the daytime direct radiative effect of aerosols, T_max_ was chosen as the surface temperature indicator.

To complement and validate the satellite-derived estimates, reanalysis data from the MERRA-2 archive (https://disc.gsfc.nasa.gov/) were used. These included AOD from M2T1NXAER (Version 5.12.4, 8:30 UTC), which integrates multiple observational sources such as AVHRR, AERONET, MISR, and MODIS^79,80^, and Tmax from M2SDNXSLV (Version 5.12.4). Additional variables included total and low cloud fraction from M2T1NXRAD (8:30 UTC) and total column water vapor from M2I1NX1NT (Mean of 8:00 UTC and 8:30 UTC).

### Methods

#### Screening and pre-processing

To ensure reliability in the quantification of aerosol effects, the MODIS AOD data were screened for outliers by removing the highest and lowest 5th percentile values at each grid point. This step helps minimize potential cloud contamination^81^ and adjacency effects, although it should be noted that the satellite products used are already of high quality. This screening is intended to further enhance confidence in the estimates. In addition, mountainous regions were excluded from the analysis, as the relationship between aerosol optical depth and surface air temperature may be confounded by elevation-dependent effects. Grid points with an average elevation exceeding 500 m above sea level were masked using 1° × 1° elevation data from the United States Geological Survey (USGS). To focus on short-term variability and remove underlying seasonal and longer-term trends, anomalies were calculated for each variable by subtracting a seven-day moving average centered on each day. This ensures the removal of any long-term and seasonal trends in the data while enhancing the data’s short-term variability.

#### Aerosol—surface air temperature relationship calculation

The change in maximum surface air temperature (Tmax) due to the predictor variables (AOD, CFR, and TCWV) is estimated using multiple linear regression, as described in Eq. 1. At each grid point, anomalies of AOD, CFR, and TCWV are regressed against the anomalies of Tmax to quantify the effect of each variable on T_max_ and statistical significance is calculated using t-test. Prior to performing the regression analysis, multicollinearity among predictors was evaluated using variance inflation factor (VIF) analysis. Across all grid points, VIF values were found to be less than 2, indicating minimal risk of collinearity between the predictor variables (Supplementary Fig. S7). The regression analysis is performed separately for each season (DJF, MAM and SON) to find the seasonal differences in aerosol effect on surface air temperature.

The regression coefficients (slopes) represent the sensitivity of T_max_ to a unit change in each predictor variable. To estimate the mean temperature change due to aerosols, the slope of the aerosol term is multiplied by the seasonal mean value of AOD at each grid point (Eq. 2), resulting in spatial maps of the estimated temperature response for each season. This approach has been previously applied by Sarin et al.^31^ to assess aerosol impacts on surface temperature. To assess model robustness, we performed an out of sample analysis using odd years as training data and even years as test data. Regression models were trained at each 31 × 31 grid point using odd years, with AOD, cloud fraction, and water vapor anomalies as predictors of Tmax anomalies, and then validated on even years. Model performance, evaluated using normalized MAE (nMAE), typically ranged between 10 and 20% of the observed variability. While regional differences in skill were noted, the predictors consistently captured a substantial fraction of Tmax anomaly variability (Supplementary Fig. S2).

1 $$\Delta {T}_{max }=(\frac{\partial {T}_{ma{x}}}{\partial AOD}) \text{x }\Delta AOD+(\frac{\partial {T}_{ma{x}}}{\partial CFR}) \text{x }\Delta CFR + (\frac{\partial {T}_{ma{x}}}{\partial TCWV}) \mathrm{x} \Delta TCWV+\mathrm{C}$$

2 $$Aerosol effect =(\frac{\partial {T}_{ma{x}}}{\partial AOD}) \text{x } AO{D}_{mean}$$

The seasonal analysis was also averaged over regions of India (Fig. 1b), representing distinct aerosol regimes: Southern India (SI), Northwest (NW), North India (NI), and Northeast India (NE). To validate the findings, the same analysis was also performed using MERRA-2 reanalysis data.

The aerosol effect presented here corresponds to the instantaneous aerosol direct or semi-direct effects, as indirect effects resulting from aerosol-induced cloud changes are integrated into the cloud fraction term and thereby minimized. The relationship between aerosols and surface air temperature is modulated by underlying land surface characteristics, including albedo and soil moisture, which generally vary more slowly than atmospheric parameters. To evaluate their impact, albedo and soil moisture from MERRA2 were incorporated into the analysis (Supplementary Fig. S3). The results show minimal changes in the estimated aerosol effect, indicating that the main findings are robust to variations in land surface properties. This also suggests that the approach used to remove the seasonal cycle effectively minimizes the potential influence of slowly varying land surface characteristics.

Given the coarse spatial resolution of 1° × 1°, the aerosol effects reported here represent an average over multiple land surface types within each grid cell for the respective season. While meteorological parameters like relative humidity (RH) and wind speed may also impact the aerosol-surface air temperature relationship, a recent study by Jangid et al.^48^ demonstrated that these factors have a minimal effect. Therefore, they were omitted from the current analysis.

#### RegCM model set-up and experiments

The present study employs version 4.7.1 of the Abdus Salam International Centre for Theoretical Physics (ICTP) Regional Climate Model, RegCM4.7.1^82^. This hydrostatic model utilizes a sigma-p vertical coordinate system and a dynamical core adapted from the NCAR MM5 model. Radiative transfer calculations are based on the Community Climate Model version 3 (CCM3). RegCM4 has been widely applied to investigate aerosol and meteorological processes over India^69,83–88^. The configuration of the current study has been done following^63^.

The model incorporates the Community Land Model version 4.5 (CLM4.5^66^) for land surface processes, which has demonstrated strong performance in simulating regional climate over India^89–91^. Planetary boundary layer dynamics are represented by the Holtslag scheme^92^. Convection is parameterized using the Grell scheme over oceans^93^ and the Emanuel scheme over land^94^. Large-scale cloud and precipitation processes are simulated using the SUBEX scheme^95^.

RegCM4.7.1 is coupled with an online aerosol module that accounts for both natural and anthropogenic aerosols. The anthropogenic components include sulfate, black carbon (BC), and organic carbon (OC)^96^. The module simulates four size bins of dust^97^, two bins of sea salt^98^, hydrophobic and hydrophilic fractions of BC and OC, and aqueous-phase sulfate formation, resulting in a total of 12 tracers. Aerosols are removed via dry and wet deposition processes. Notably, the model includes only the direct radiative effects of aerosols; indirect effects through cloud microphysics are not considered. The direct effect influences atmospheric temperature tendencies, which in turn modulate thermodynamic, meteorological, and radiative fields.

Anthropogenic emissions for SO₂, BC, and OC are sourced from the ECLIPSE v5a inventory (International Institute for Applied Systems Analysis; https://iiasa.ac.at/web/home/research/researchPrograms/air/ECLIPSEv5a.html) for the period 2007–2008. Simulations were conducted at a horizontal resolution of 50 km with 18 vertical sigma levels, extending up to 50 hPa. The model domain spans 0–45°N and 55–110°E, with results analyzed for the Indian region (67.5°E–97.5°E and 7.5°N–37.5°N). Initial and lateral boundary conditions were taken from the ERA-Interim reanalysis, while weekly optimal interpolated sea surface temperatures (1° × 1° resolution) were obtained from NOAA. Surface elevation data are based on USGS topography.

The model was run from 1 November 2007 to 30 November 2008, with the first month used as a spin-up. Two experiments were conducted: (1) a control run (CTRL) without aerosol–radiation interactions, and (2) a perturbed experiment (EXP) in which aerosols influenced the radiation field through the direct effect. The aerosol-induced surface air temperature response was quantified as:

3 $${\mathrm{AER}}\_{\mathrm{EFF}}_{{{\mathrm{MOD}}}} = {\text{ T}}_{{{\mathrm{max}}\_{\mathrm{EXP}}}} {-}{\mathrm{T}}_{{{\mathrm{max}}\_{\mathrm{CTRL}}}}$$

Although multi-year simulations would provide stronger statistical robustness, this single-year simulation is designed as an idealized study to isolate aerosol-radiation effects and understand the physical mechanisms driving aerosol-SAT interactions while minimizing computational expense. Future work can extend this framework to multi-year simulations to capture interannual variability in the aerosol effect.

#### Calculation of the effect of clouds

Daily-averaged, gridded daytime CTP data at 1° × 1° resolution were used to classify cloud types based on the International Satellite Cloud Climatology Project (ISCCP) criteria^99^. According to ISCCP, clouds are categorized into nine types using CTP and cloud optical thickness. For this study, only the CTP-based classification was used, where clouds with CTP > 680 hPa are identified as low-level clouds.

To assess how low clouds respond to aerosol loading, the CTP data were binned based on AOD values. Two subsets were created: one corresponding to the top 20th percentile of AOD values (high-aerosol days) and the other to the bottom 20th percentile (low-aerosol days). For each subset, the number of days classified as low cloud days was counted based on the corresponding CTP values. The difference in the frequency of low-cloud days between high and low AOD conditions was used as a proxy to examine the aerosol influence on low cloud occurrence (Eq. 4).

4 $$\Delta LC{D}_{OBS}=LC{D}_{AOD\ge 80th}-LC{D}_{AOD\ge 20th}$$

The resulting patterns were then compared with the AER_EFF_OBS_ to understand whether aerosol effects on surface air temperature are mediated through changes in low cloud cover. The $\Delta$ LCC for MERRA2 was calculated similarly using the low cloud cover through Eq. 5 and then compared the same way with AER_EFF_RA_.

5 $$\Delta LC{C}_{RA}= {LC{C}_{AOD\ge 80th}} - {LC{C}_{AOD\ge 20th}}$$

In the RegCM simulations, cloud fraction at low level is available as a model output. The difference between the aerosol experiment (EXP) and the control simulation (CTRL) provides the change in cloud fraction due to aerosol forcing (Eq. 6) and then compared with AER_EFF_MOD_.

6 $$\Delta LC{C}_{MOD}= {LC{C}_{EXP}}-{LC{C}_{CTRL}}$$

To examine the relationship between cloud changes and the sign of aerosol impact on temperature, grid points were separated into two categories based on whether AER_EFF_MOD_ was positive or negative. For each category, the mean change in cloud fraction at each level was computed (Fig. 3a,b).

## Supplementary Information

Below is the link to the electronic supplementary material.

Supplementary Material 1

## Data Availability

All the data utilised for this study are publicly available. The details are provided in the data section. The analysed data from the current study is available from the corresponding author on reasonable request.

## Code availability

All analyses were done using MATLAB (version R2022b; https://www.mathworks.com). The custom codes used for data preprocessing, anomaly calculation, regression analysis, and figure generation in this study are available from the corresponding author upon reasonable request.
