# Supplementary material for: Assessing the relationship between atmospheric aerosols and maximum surface air temperature over the Indian region
Source: Sci Rep. 2026 Feb 18;16:9483. doi: 10.1038/s41598-026-40641-0 (PMC13004928; doi:10.1038/s41598-026-40641-0)
Supplement: Supplementary file 1 — Supplementary Material 1 [file 41598_2026_40641_MOESM1_ESM.docx]

**The Relationship between Atmospheric Aerosols and Maximum Surface Air Temperature over the Indian Region**

Sarin. T. S, V. Vinoj

School of Earth, Ocean, and Climate Sciences

Indian Institute of Technology Bhubaneswar (IIT BBS), Argul, Odisha, INDIA 752050

Correspondence email: vinoj@iitbbs.ac.in

**Supplementary Materials**

**S1: Model (RegCM) Simulations to Explore the Aerosol Effect**

**
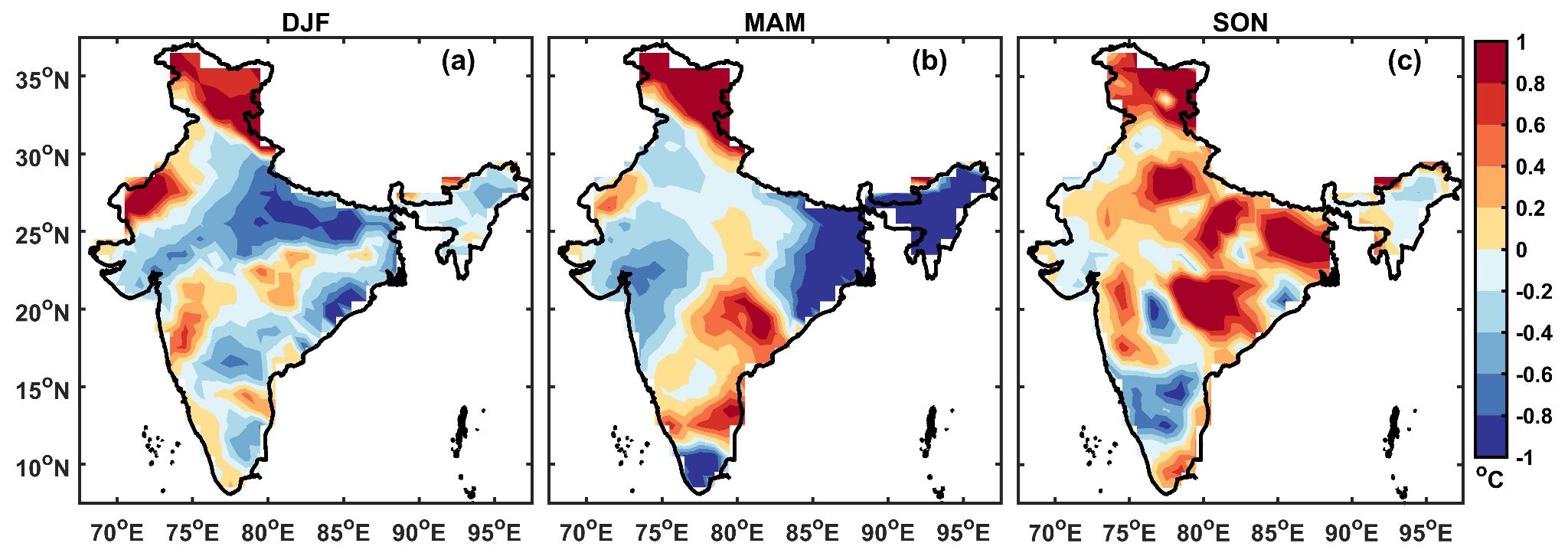
**

**Figure S1:** Spatial pattern of surface temperature due to aerosols during (a) DJF, (b) MAM, (c) SON for 2007 December to 2008 November, calculated using RegCM 4.7.1

Fig. S1 presents the spatial distribution of the aerosol effect on surface temperature over India, simulated using the RegCM 4.7.1 model (Giorgi et al., 2012) coupled with CLM 4.5 ( Oleson et al., 2010), for the years 2007–2008. During the DJF season (Fig. S1a), the aerosol effect is predominantly negative, with maximum cooling of ~1°C concentrated over the Indo-Gangetic Plains. In contrast, isolated warming of ~1°C is observed over the Thar Desert and mountainous regions in the north. The warming over the Thar Desert may be attributed to radiative heating of the lower atmosphere or the burn-off of clouds caused by the high dust concentration. Meanwhile, warming in the mountainous regions could result from the deposition of absorptive aerosol particles, such as dust, on snow surfaces, which reduces albedo and increases sensible heat flux, leading to further warming (Nair et al., 2024; Painter et al., 2018).

During the MAM season, regions in Northern India experiencing negative aerosol effects shift eastward, with maximum cooling of ~1°C. Moderate warming is observed over the Deccan Plateau, while significant warming occurs in the Himalayan region, potentially due to increased dust transport characteristic of the season. By SON, the spatial pattern changes, with high aerosol-induced warming at the surface. This warming pattern is notably distinct from observation-based studies, which report a warming effect during the MAM season. Significant biases persist in aerosol simulations over India using RegCM (Ajay et al., 2019), particularly in representing anthropogenic aerosols, due to the unavailability of high-quality emission inventories (Lamarque et al., 2010; Boucher et al., 2013). However, despite discrepancies in spatial and temporal patterns, the model successfully simulates both the warming and cooling effects of aerosols on maximum surface temperature. This suggests that analysing regions with distinct negative and positive aerosol effects could offer insights into the mechanisms driving aerosol-induced surface temperature changes in observational data.

The aerosol radiative effect at the surface is negative, ruling out the direct effect as the sole factor in causing localised positive effects on surface air temperature in certain regions. However, it adequately explains negative aerosol effects in others. This suggests that aerosols also modulate surface temperature through interactions with cloud properties and coverage across multiple atmospheric levels via the semi-direct effect (Hansen et al., 1997; Koch and Del Genio, 2010). This process, which can either enhance or inhibit convection and cloud formation, may play a role as significant as the direct and indirect effects in shaping surface temperature patterns.

### **S2: Validation of the Aerosol–Temperature Relationship**

An out-of-sample validation to assess the predictive skill of the multiple linear regression model relating maximum surface air temperature anomalies (T_max_) to aerosol optical depth anomaly (AOD), cloud fraction anomaly (CFR), and cloud water content anomaly (TCWV). The analysis was conducted at each 31 × 31 grid point. The dataset was divided into odd years (training set) and even years (test set). The regression coefficients were estimated using the training set and subsequently applied to the test set. In our study, we have selected the Mean Absolute Error (normalised by the range of the Tmax anomaly at each grid point and expressed as a percentage), a widely used error metric for evaluating model performance. The normalisation of the MAE allows comparison of the error obtained.

nMAE quantifies the average magnitude of prediction errors without considering their direction. The normalisation by the data range enables spatial comparison over regions with varying climatic conditions and helps us understand how much the percentage error is when compared to the variability of data in that grid point. The normalisation is done using the range of the true values rather than the mean, as the anomaly values may cancel each other out, leading to mean values being close to zero and the error percentage blowing up. nMAE is always positive, and a low nMAE value close to zero is desired.

$nMAE (\%)=$ $\frac{100}{n}$ $\frac{\sum{}_{i=1:n} |y_{t,i}-y_{p,i}|}{Range (y_{t})}$ →(S1)


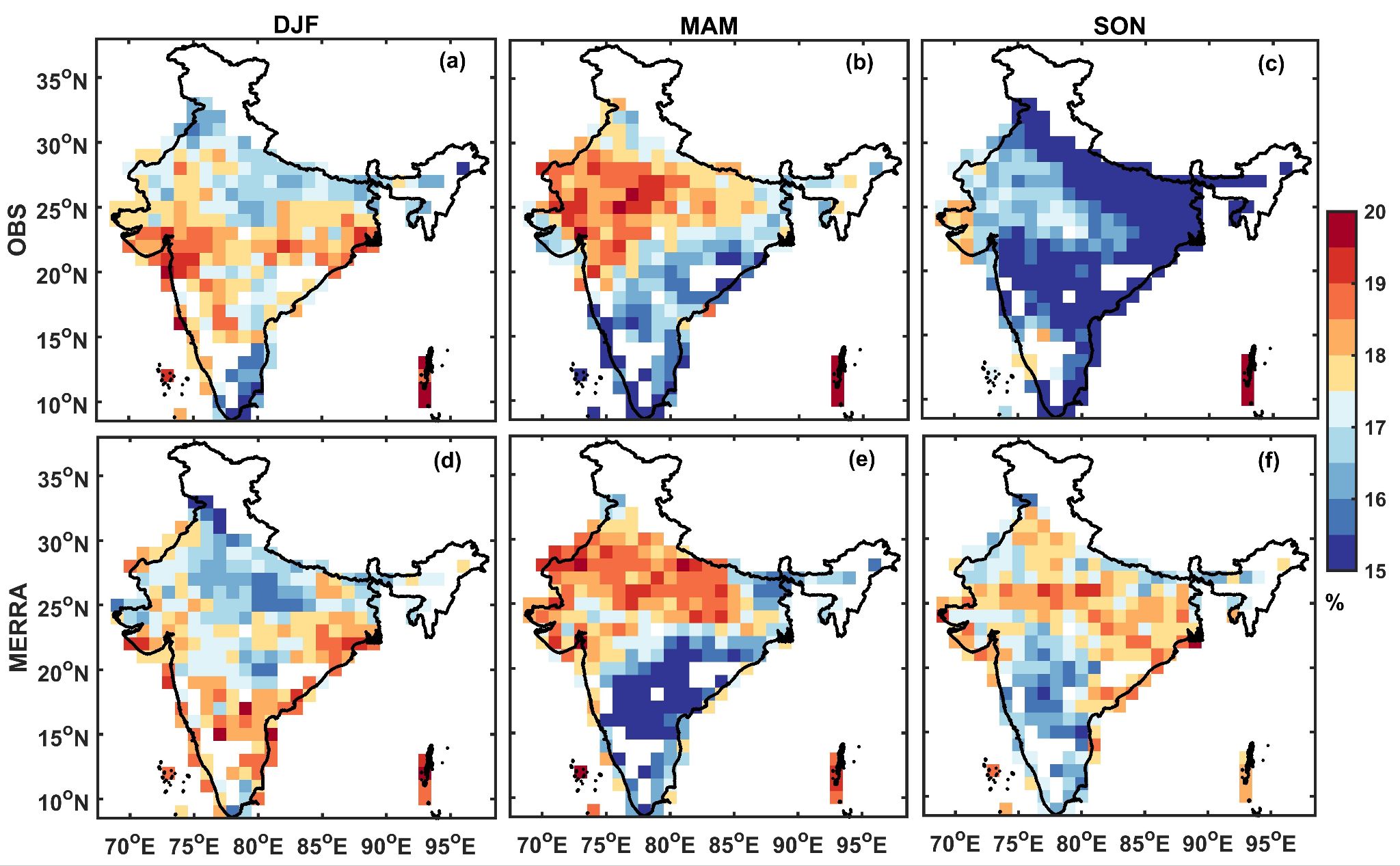


**Figure S2:** Spatial pattern of nMAE (a) DJF, MODIS (b) MAM, MODIS (c) SON, MODIS (d) DJF, MERRA (e) MAM, MERRA (f) SON, MERRA

The error values are in the range of 10-20% over much of India during all seasons over the Indian region, which could be considered as a fair prediction of the T_max_ anomaly. We can also see a clear spatial pattern in the model errors. Regions with higher temperatures show greater nMAE compared to other regions. This is evident over SI during DJF (Fig. S2 (a))and over NW and NI during MAM (Fig. S2 (b)). This behaviour can also be seen over the NW during SON as well. Higher variability and extremes in temperature are linked to strong convective activity and local land atmospheric processes, which introduce non-linearities that are harder to model accurately using a linear framework. We also note that nMAE is systematically higher when the model is validated against MERRA-2 test data than when validated against MODIS test data. This discrepancy is attributed to differences between the datasets, where MERRA2 fields are reanalysis products produced by a model assimilation system, while MODIS variables are direct satellite retrievals at Aqua overpass times.

### **S3: Sensitivity of Aerosol-Temperature Relationship to Land Surface Parameters**


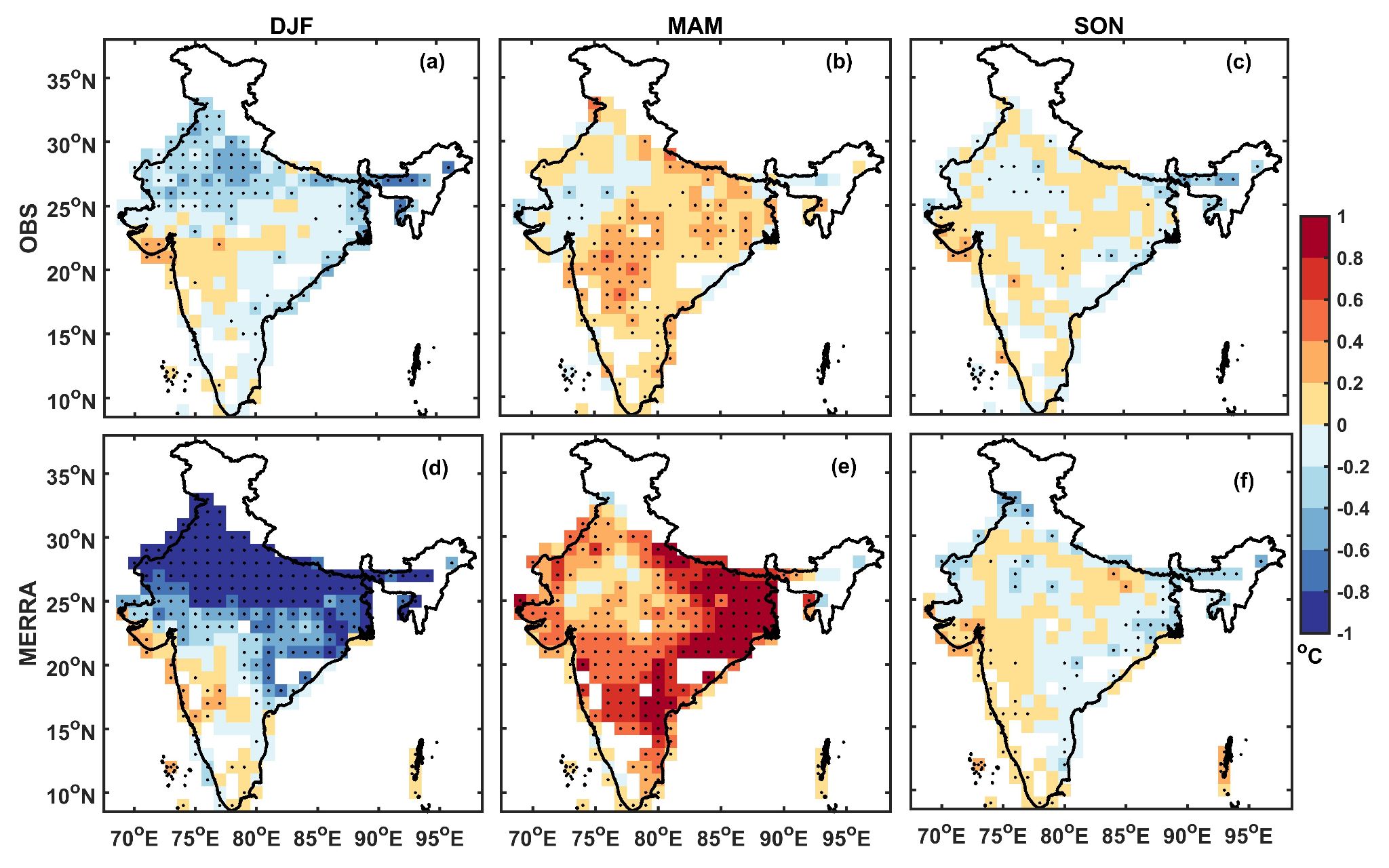


**Figure S3.** Aerosol effect estimated with albedo and soil moisture effects for (a) MODIS, DJF, (b) MODIS, MAM, (c) MODIS, SON, (d) MERRA, DJF, (e) MERRA, MAM, (f) MERRA, SON,

To examine whether land surface characteristics influence the estimated aerosol-temperature relationship, we incorporated MERRA2 soil moisture (0-5cm; M2T1NXLND 5.12.4; 8:30 UTC) and surface albedo (M2T1NXRAD 5.12.4; 8:30 UTC) into the regression framework. Data from September 2002 to 2024 were used, and the multi-linear regression was repeated with these parameters included as additional independent variables alongside aerosol optical depth (AOD) anomaly, cloud fraction anomaly, and total column water vapour anomaly.

The results show that including land surface variables slightly alters the magnitude of aerosol effects in MERRA-based calculations, particularly over northwestern India, while the spatial patterns remain largely unchanged. In contrast, aerosol effects derived from observational datasets are minimally affected by the inclusion of these variables. This limited impact likely reflects the slower temporal variability of land surface characteristics relative to anomalies in temperature and aerosol loading, coupled with the seasonal cycle removal method, which emphasises short-term variability and diminishes the influence of slowly varying surface properties. Overall, these findings indicate that the observed aerosol–temperature relationships predominantly reflect aerosol-driven effects rather than being substantially confounded by land surface variability.

### **S4: AOD Spatial Distribution and Model Biases over India for Three Seasons (RegCM, MODIS, MERRA2)**

Fig. S4 shows the spatio-temporal distribution of seasonal AOD simulated by RegCM at 350–640 nm (bottom row), MERRA2 AOD at ~ 550 nm (middle row) and MODIS AOD (top row) over the Indian Subcontinent. A direct comparison between simulated AOD in the visible band (350–640 nm) and MODIS AOD at 550 nm is not strictly possible due to the difference in wavelengths. However, a similar comparison of seasonal AOD bias, following the approach of Ajay et al. (2019), yields consistent patterns in magnitude and spatial patterns. Spatio-temporal AOD variation is well depicted by the model, like the simulation of AOD hot spots like IGP during DJF associated with anthropogenic emission, high AOD over Northern India during MAM during dust activities and the washout of aerosols during the monsoon season.


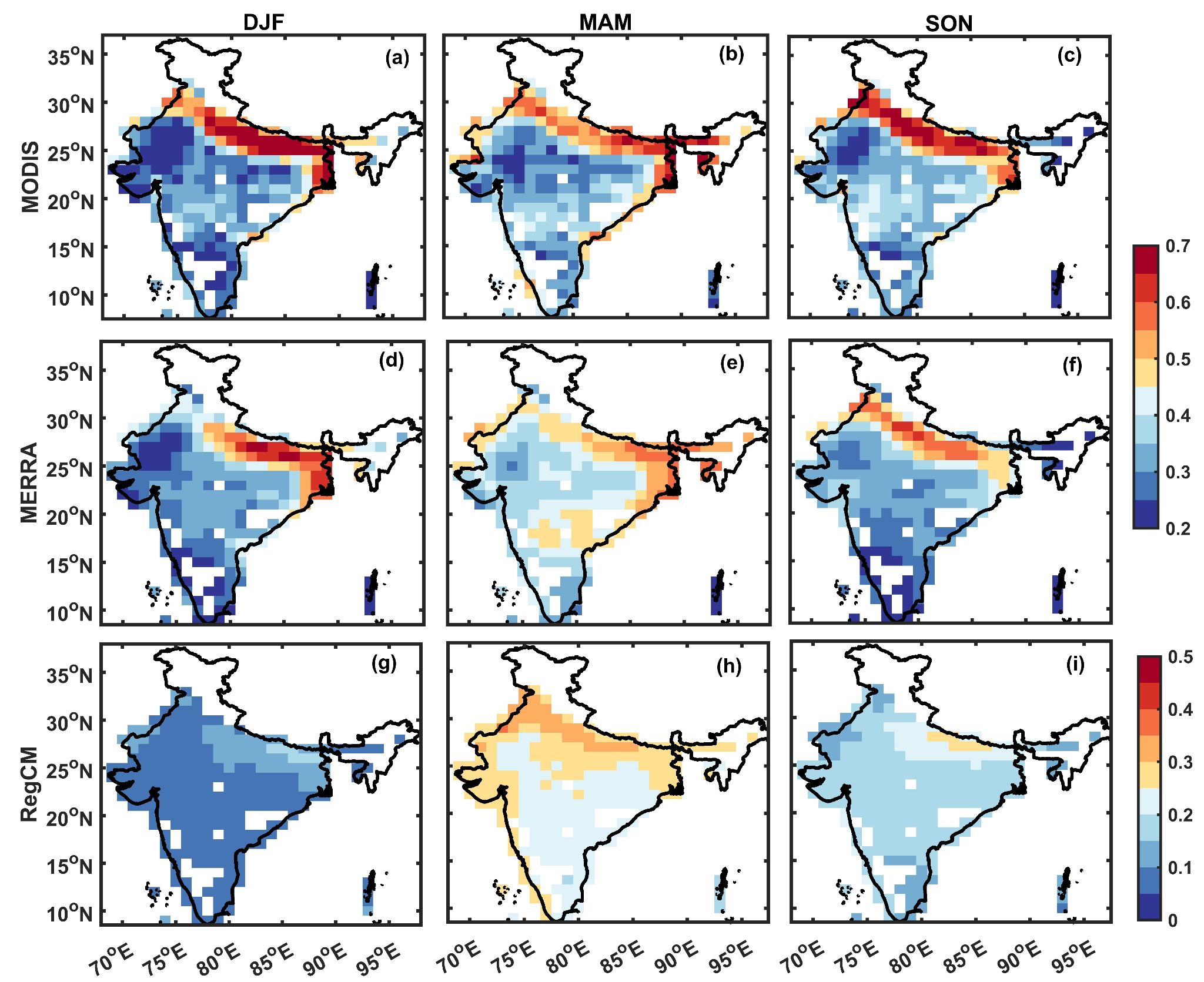


**Figure S4:** Spatial distribution of AOD: (a) MODIS, DJF; (b) MODIS, MAM; (c) MODIS, SON; (d) MERRA, DJF; (e) MERRA, MAM; (f) MERRA, SON; (g) RegCM, DJF; (h) RegCM, MAM; (i) RegCM, SON. Panels (a–f) share a common colorbar, while panels (g–i) use a separate colorbar to better illustrate spatial variations.


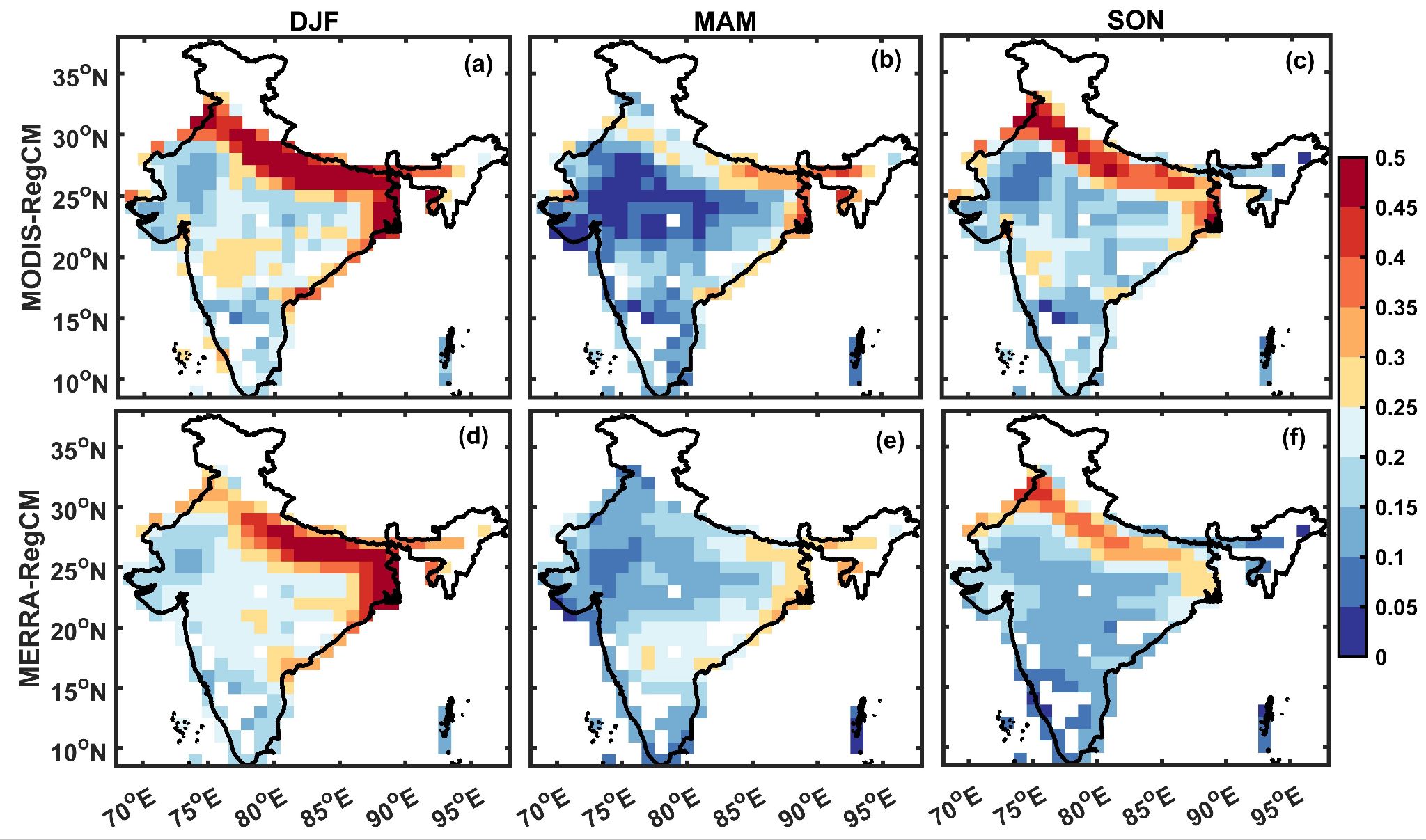


**Figure S5:**Spatial distribution of seasonal AOD bias simulated by RegCM compared with (a) MODIS, DJF, (b) MODIS, MAM, (c) MODIS, SON, (d) MERRA, DJF, (e) MERRA, MAM, (f) MERRA, SON

The largest bias occurs over the Indo-Gangetic Plain (IGP) during winter (DJF), where RegCM underestimates MODIS AOD by approximately 0.5 and is followed by the post-monsoon season (Fig. S5). This underestimation is primarily attributed to limitations in emission inventories, which leads to DJF AOD being underestimated in areas dominated by anthropogenic aerosol sources relative to natural aerosols. Similar underestimation over India during winter has been reported in earlier studies (Nair et al., 2012), highlighting challenges in accurately representing emissions and atmospheric chemistry. Simulated and satellite-retrieved AOD show the closest agreement during the pre-monsoon (MAM) season, with biases generally within ±0.2. RegCM captures the northwesterly transport of mineral dust effectively. The model also captures the lower AOD during SON due to rain washout, and biases slowly start increasing during SON.

**S5: Regional contrasts in aerosol-low-cloud relationships over India**

To understand the regional differences of aerosol-low cloud relationships over India and their relationship to the aerosol effect on temperature, we extended the analysis underlying Fig. 3(f) by stratifying ΔLCD_OBS_ and ΔLCD_RA_ by region and by the sign of AER_EFF_OBS_ and AER_EFF_RA,_ respectively.

**Table S1:** Regional aerosol-low-cloud relationships by AER_EFF_OBS_ regime (rounded off to nearest whole number)

|  | **AER_EFF_OBS_ >0** | **AER_EFF_OBS_ <0** | **AER_EFF_RA_ >0 (%)** | **AER_EFF_RA_ <0 (%)** |
| --- | --- | --- | --- | --- |
| **SI** | 9 | 35 | -1.60 | 14.44 |
| **NW** | 45 | 62 | -30.22 | -1.86 |
| **NI** | 38 | 41 | -6.28 | -8.35 |
| **NE** | 32 | -1 | -26.92 | -3.10 |
| **All India** | 30 | 45 | -15.08 | 0.59 |

The enhancement in ΔLCD_OBS_ when AER_EFF_OBS_ < 0 °C is most pronounced over northwestern India (NW), with a smaller increase over northern India (NI). Both these regions are characterised by high aerosol loading, where radiative effects can stabilise the lower troposphere and favour low-cloud occurrence during DJF and absorbing aerosols during MAM can lead to cloud burn off. The NI region could show a muted effect due to the competing effects of both scattering and absorbing aerosols abundant in the region (Shahid et al., 2022). In contrast, the northeast (NE) shows a decrease in ΔLCD_OBS_, while southern India (SI) exhibits a large increase, despite lower aerosol loading compared to NW and NI. This suggests that regional meteorological processes may also contribute, and that observational analysis alone cannot fully disentangle aerosol effects from meteorological influences, instead providing a view of their combined impact. Overall, India shows an increase of ~15 low-cloud days under AER_EFF_OBS_ < 0 °C compared to > 0 °C, indicating that part of the aerosol-temperature effect may be mediated through aerosol-induced changes in low cloud cover.

The analysis using AER_EFF_RA_ and ΔLCD_RA_ gives a clearer picture of ΔLCD_RA_ being more negative when AER_EFF_RA_ is greater than zero, compared to when AER_EFF_RA_ is less than zero over all regions. To minimise the influence of meteorological variability and better isolate aerosol effects, RegCM simulations were examined. These reveal a consistent negative relationship between ΔLCC_MOD_ and AER_EFF_MOD,_ further proving that aerosol-SAT relationships are mediated by changes in the low cloud cover.

**S6: Temporal Evolution of Aerosol Effects Across Seasons during two epochs (2002-13 & 2014-24)**

To assess whether the observed aerosol effects have changed over time, the study period was divided into two epochs: 2002–2013 and 2014–2024. Regression analyses conducted separately for these periods reveal spatial patterns broadly consistent with those obtained using the full record, thereby confirming the robustness of the main findings. However, the magnitude of the effects has strengthened in the latter half, suggesting that aerosol–temperature interactions have become more pronounced in recent years. During DJF, enhanced cooling is observed, particularly over northwestern India and the northeastern region, with mean decreases of ~0.1–0.2°C relative to the earlier epoch. In MAM, warming has intensified over the Indo-Gangetic Plain and central India, although a reduction is noted over the northeast. In contrast, SON exhibits a widespread increase in cooling in the later period compared to the earlier one.

**
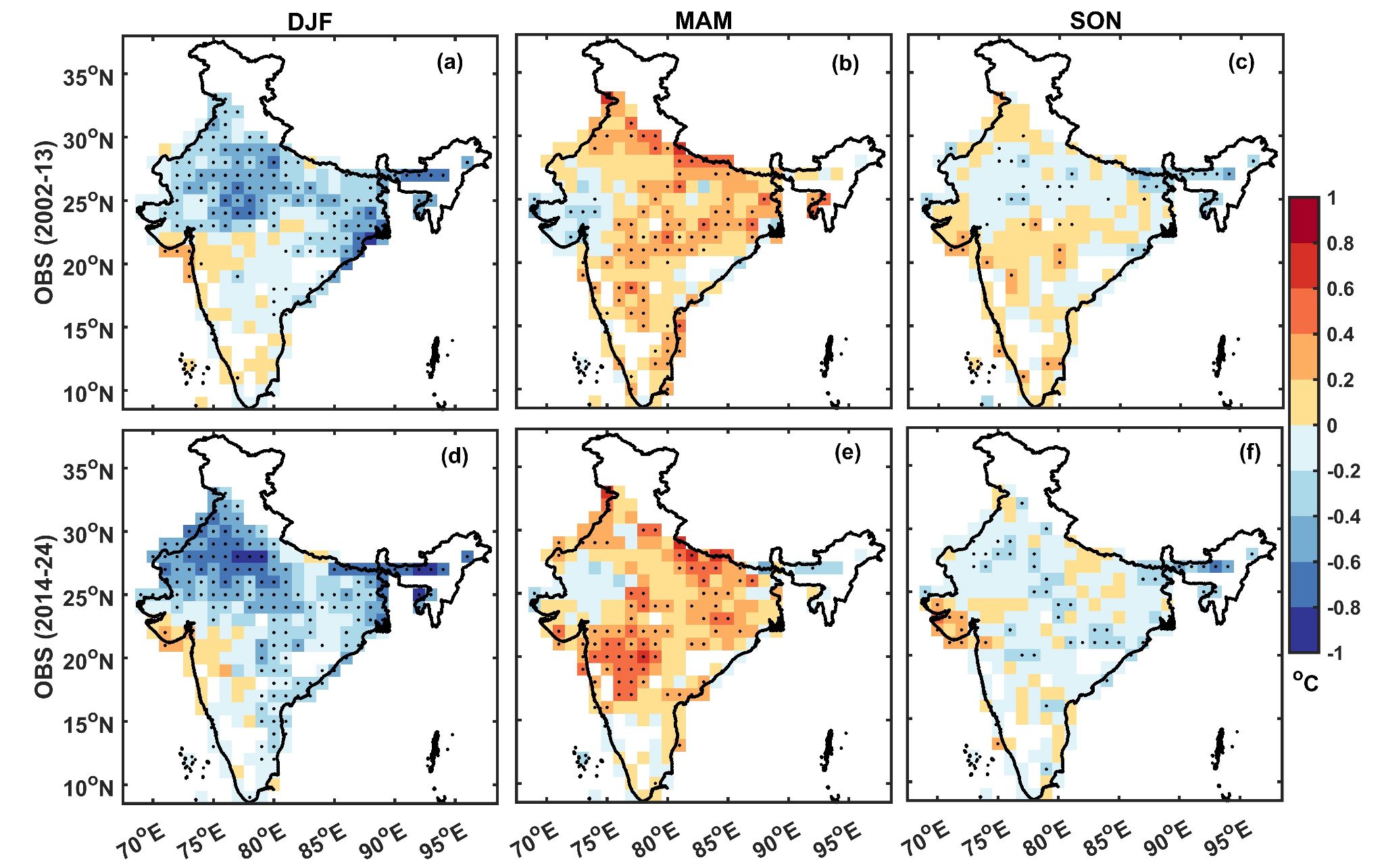
**

**Figure S6**: Aerosol effect estimated (a-c) using observational data (2002-13) for DJF, MAM, and SON, respectively; and (d-f) using observational data (2014-24) for DJF, MAM, and SON, respectively.

These results indicate that the persistence of the aerosol effects is not merely an artefact, but reflects a temporally robust and increasingly evident signal. While a detailed attribution is beyond the scope of the present study, several factors may contribute, including evolving emission patterns (e.g., changes in biomass burning, dust mobilisation, and black carbon emissions), regional shifts in land-atmosphere feedbacks, and modulation by large-scale circulation variability. Together, these findings demonstrate that the aerosol–temperature relationship in India is not only consistent across time but is also intensifying, underscoring the importance of sustained monitoring and further investigation.

**S7: Multicollinearity Assessment Using Variance Inflation Factor (VIF)**

To ensure that the estimated effect of aerosols on surface air temperature is not influenced by collinearity with other predictor variables, we performed a multicollinearity diagnostic using the Variance Inflation Factor (VIF). The regression model includes aerosol optical depth (AOD) as the primary predictor, with cloud fraction and total column water vapour (TCWV) serving as potential confounding covariates. The VIF was computed as:

$VIF =$ $1/1- R^{2}$ →(S2)

where $R^{2}$ corresponds to the coefficient of determination obtained by regressing the AOD anomaly against the CFR and TCWV anomalies.


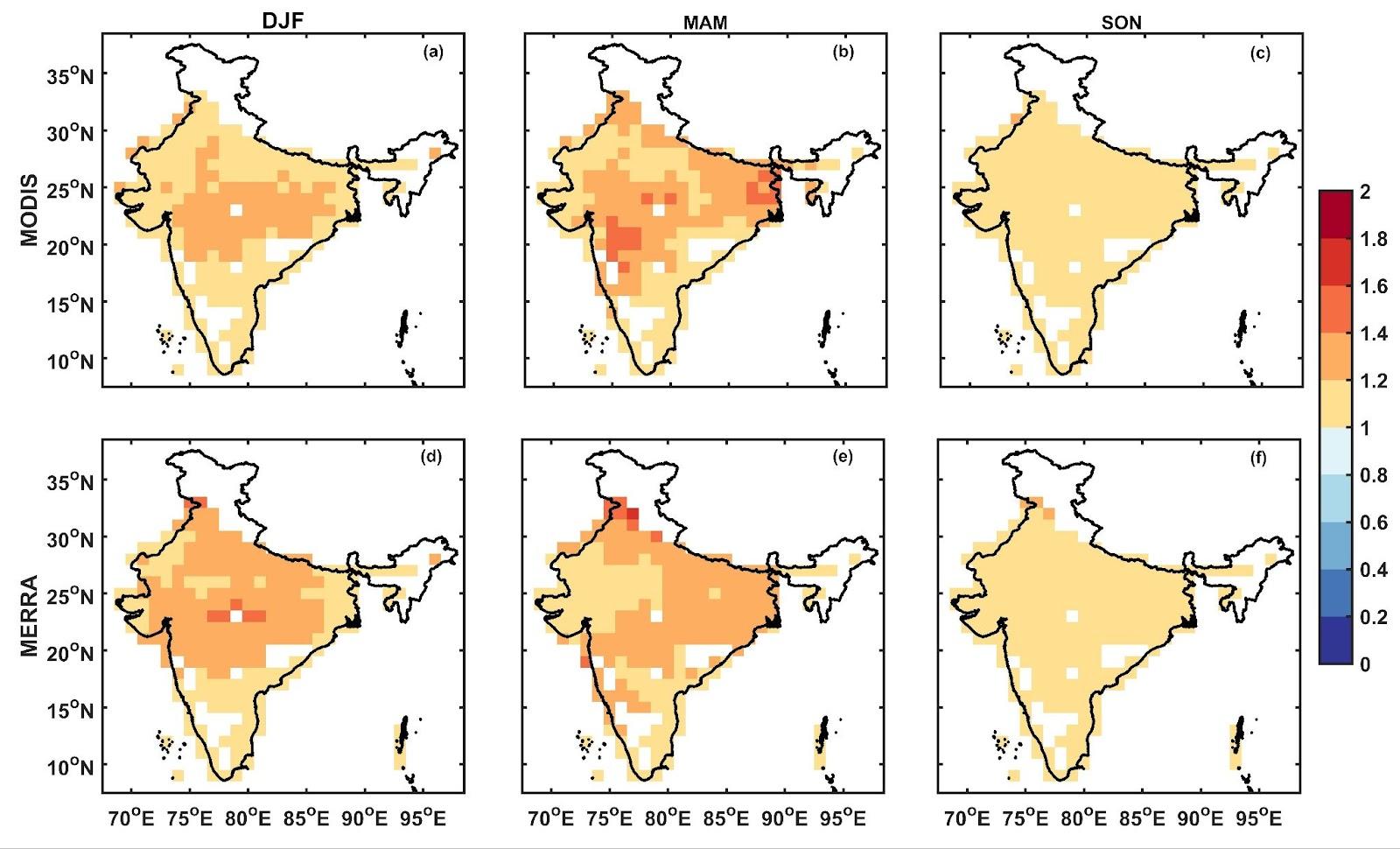


**Figure S7**: Variance Inflation Factor (VIF) for deseasonalised AOD estimated (a-c) using observational data for DJF, MAM, and SON, respectively; and (d-f) using MERRA2 data for DJF, MAM, and SON, respectively.

The resulting VIF for AOD was found to be below 2 for all grid points, well within the generally accepted thresholds indicating low multicollinearity (VIF < 5: acceptable; VIF < 2: minimal collinearity). This confirms that the regression estimates can be interpreted with confidence. The VIF of other variables were similarly calculated and were found to be less than 2.
